# Comment on "Could signal peptide complement C1r/C1s, Uegf, and Bmp1, and epidermal growth factor-containing protein 1 be a therapeutic target in the pathogenesis of preeclampsia?"

**DOI:** 10.1590/1806-9282.20250691

**Published:** 2025-10-17

**Authors:** Chen Cheng, Yanxiang Wang

**Affiliations:** 1 Yangzhou Polytechnic College, Department of Medical Science – Yangzhou, China.; 2 Haimen People's Hospital, Department of Gynecology and Obstetrics – Haimen, China.

Dear Editor,

We have read with great interest the article entitled "Could signal peptide complement C1r/C1s, Uegf, and Bmp1, and epidermal growth factor-containing protein 1 be a therapeutic target in the pathogenesis of preeclampsia?" by Toprak et al.^1^. Preeclampsia (PE), a common complication of pregnancy, may lead to maternal death in severe cases. Therefore, it is of great significance to develop an indicator for predicting PE. We give praise to the authors for their valuable contributions. They have found that signal peptide complement C1r/C1s, Uegf, and Bmp1, and epidermal growth factor-containing protein 1 (SCUBE1) is an independent predictor for PE. Nevertheless, certain concerns outlined below warrant additional clarification.

First, this study lacks the inclusion of non-pregnant healthy women. A total of 73 healthy pregnant women were included in this study as a control group, and it was found that the serum level of SCUBE1 in pregnant women with PE was significantly higher than that in healthy pregnant women. However, the expression of SCUBE1 in non-pregnant healthy women has not been reported. In this sense, two hypotheses may be proposed. First, SCUBE1 may be expressed at low levels in non-pregnant healthy women, with expression increasing after pregnancy and further increasing in cases of PE. Under this hypothesis, the high expression of SCUBE1 is not only related to the pathological state of PE but also to pregnancy itself. Second, the expression level of SCUBE1 in non-pregnant healthy women is not significantly different from that in healthy pregnant women. Under this hypothesis, serum SCUBE1 is closely related to the risk of PE and has greater clinical significance. Therefore, in the absence of a non-pregnant healthy women group, the clinical significance of serum SCUBE1 is not yet clear.

Second, previous studies have confirmed that C-reactive protein (CRP) increases during PE^2^ and is associated with adverse maternal outcomes^3^. In this study, the CRP level in the PE group was significantly higher than that in the healthy group, so it is recommended to include CRP in both univariate and multivariate regression analyses to strengthen the conclusion that SCUBE1 is an independent risk factor for PE. In addition, CRP should be included in the receiver operating characteristic (ROC) curve analysis to determine whether the predictive value of SCUBE1 for PE is greater than that for CRP.

Third, the authors have listed a history of diabetes as an exclusion criterion, but diabetes in the history differs from that in the gestational period. The American Diabetes Association defines gestational diabetes as diabetes first diagnosed in the second or third trimester of pregnancy, with no history of diabetes before pregnancy^4^. Liu et al. found that the expression of SCUBE1 in the placental tissue of patients with gestational diabetes was significantly higher than that in healthy pregnant women^5^. Bayoglu Tekin et al. confirmed that the expression of plasma SCUBE1 in patients with gestational diabetes was significantly higher than that in healthy pregnant women^6^. If some of the PE patients in this study had gestational diabetes, it might have biased the results.

Finally, there was a difference in serum creatinine between the healthy pregnant women group and the PE group (p=0.016), but there was no difference in blood urea nitrogen (p=0.101). It is well established that blood urea nitrogen and serum creatinine are classic indicators of renal function^7^; however, the profiles of both indicators showed inconsistency between groups. If the difference in estimated glomerular filtration rate (eGFR) between the two groups is analyzed, the influence of renal function on PE may be ruled out.

## Data Availability

The datasets generated and/or analyzed during the current study are available from the corresponding author upon reasonable request.
